# Muscle-specific loss of *Bmal1* leads to disrupted tissue glucose metabolism and systemic glucose homeostasis

**DOI:** 10.1186/s13395-016-0082-x

**Published:** 2016-03-30

**Authors:** Brianna D. Harfmann, Elizabeth A. Schroder, Maureen T. Kachman, Brian A. Hodge, Xiping Zhang, Karyn A. Esser

**Affiliations:** 1 Center for Muscle Biology, University of Kentucky, Lexington, KY USA; 2 Department of Physiology, College of Medicine, University of Kentucky, Lexington, KY USA; 3 Department of Internal Medicine, University of Michigan, Ann Arbor, MI USA; 4 Present address: Department of Physiology and Functional Genomics, Myology Institute, University of Florida, Gainesville, FL 32610-0274 USA

**Keywords:** Glucose metabolism, Skeletal muscle, Circadian rhythm

## Abstract

**Background:**

Diabetes is the seventh leading cause of death in the USA, and disruption of circadian rhythms is gaining recognition as a contributing factor to disease prevalence. This disease is characterized by hyperglycemia and glucose intolerance and symptoms caused by failure to produce and/or respond to insulin. The skeletal muscle is a key insulin-sensitive metabolic tissue, taking up ~80 % of postprandial glucose. To address the role of the skeletal muscle molecular clock to insulin sensitivity and glucose tolerance, we generated an inducible skeletal muscle-specific *Bmal1*^−/−^ mouse (iMS*Bmal1*^−/−^).

**Results:**

Progressive changes in body composition (decreases in percent fat) were seen in the iMS*Bmal1*^*−/−*^ mice from 3 to 12 weeks post-treatment as well as glucose intolerance and non-fasting hyperglycemia. Ex vivo analysis of glucose uptake revealed that the extensor digitorum longus (EDL) muscles did not respond to either insulin or 5-aminoimidazole-4-carboxamide ribonucleotide (AICAR) stimulation. RT-PCR and Western blot analyses demonstrated a significant decrease in mRNA expression and protein content of the muscle glucose transporter (*Glut4*). We also found that both mRNA expression and activity of two key rate-limiting enzymes of glycolysis, hexokinase 2 (*Hk2*) and phosphofructokinase 1 (*Pfk1*), were significantly reduced in the iMS*Bmal1*^*−/−*^ muscle. Lastly, results from metabolomics analyses provided evidence of decreased glycolytic flux and uncovered decreases in some tricarboxylic acid (TCA) intermediates with increases in amino acid levels in the iMS*Bmal1*^*−/−*^ muscle. These findings suggest that the muscle is relying predominantly on fat as a fuel with increased protein breakdown to support the TCA cycle.

**Conclusions:**

These data support a fundamental role for *Bmal1*, the endogenous circadian clock, in glucose metabolism in the skeletal muscle. Our findings have implicated altered molecular clock dictating significant changes in altered substrate metabolism in the absence of feeding or activity changes. The changes in body composition in our model also highlight the important role that changes in skeletal muscle carbohydrate, and fat metabolism can play in systemic metabolism.

## Background

Glucose metabolism in skeletal muscle requires regulation at multiple steps including glucose uptake, glucose storage, and glucose oxidation or glycolysis. At the cellular level, glucose is transported from the blood into the cells through glucose transporters, and once in the cell, it is either stored (glycogenosis) or oxidized (glycolysis). Normal glucose metabolism is crucial in regulating glucose homeostasis and providing energy for cells [20]. Dysfunction in glucose metabolism is strongly associated with metabolic diseases such as insulin resistance and diabetes [4, 7, 15]. These disease states have also been linked to circadian rhythms, as disruption in circadian rhythms can lead to development of metabolic disease [14, 16, 29].

Circadian rhythms are approximate 24-h oscillations in a variety of outputs including sleep/wake cycles, metabolism, and many aspects of cellular function. Circadian rhythms are generated by a transcription-translation feedback loop known as the molecular clock. Simply described, this clock has positive limb factors, brain and muscle arnt-like protein 1 (*Bmal1*) and circadian locomotor output cycles kaput (*Clock),* and negative limb genes period (period 1 and 2 (*Per*1/2)) and cryptochrome (*Cry1/2*) [17, 32]. *Bmal1* and *Clock* are transcribed and translated and heterodimerize in the nucleus of the cell. The BMAL1-to-CLOCK heterodimer then binds E-box elements in the promoter sequences of *Per1/2* and *Cry1/2* and promotes transcription. *Per1/2* and *Cry1/2* are transcribed and then translated in the cytoplasm. Then, PER1/2 and CRY1/2 dimerize/heterodimerize and translocate to the nucleus where they have an inhibitory effect on the activity of BMAL1-to-CLOCK. The inhibition is eventually lifted, however, as PER1/2 and CRY1/2 protein is degraded by E3 ubiquitin ligases. In this manner, a cycle of approximately 24 h is generated [10, 35]. BMAL1-to-CLOCK clock can also bind the E-box elements of genes outside of the molecular clock (clock-controlled genes) allowing the molecular clock to govern physiological and behavioral processes. Since the molecular clock is present in almost every cell of the body, the modulation of specific clock-controlled genes means that circadian rhythms regulate time-of-day tissue-specific functions and that disruption of the molecular clock in cells does alter cell function [24, 33, 34]. As previously stated, circadian rhythms have an important role in metabolism, and consequently metabolism is a key function regulated by the molecular clock in a tissue-specific manner [2, 13, 19, 27, 36].

A recent bioinformatics study from our lab identified 1628 circadian mRNAs in skeletal muscle with 62 % of these genes having metabolic roles. Carbohydrate metabolism was notably one of the enriched circadian metabolic functions in skeletal muscle [13]. We found that the circadian genes involved in carbohydrate metabolism were downregulated by the loss of *Bmal1*. In contrast, genes involved in lipid metabolism were significantly upregulated in this model suggesting that loss of *Bmal1* only in the skeletal muscle contributes to selective substrate utilization independent of circadian behavior. Skeletal muscle is responsible for approximately 80 % of glucose uptake in the postprandial state; therefore, it is critical for maintaining blood glucose homeostasis [6, 11]. Thus, the purpose of the current study was to use our inducible mouse model of the skeletal muscle-specific *Bmal1* knockout to test the effect of disruption of the clock in the adult mouse on tissue glucose metabolism, downstream substrate metabolomics, as well as whole body metabolic parameters.

## Methods

### Ethical approval

All experimental procedures were done in accordance with the institutional guidelines for the care and use of laboratory animals and approved by the University of Kentucky Institutional Animal Care and Use Committee.

### Animal care and use

Inducible skeletal muscle-specific *Bmal1* knockout mice were generated as previously described [13]. We obtained the skeletal muscle-specific tamoxifen inducible Cre recombinase mouse from the Center for Muscle Biology at the University of Kentucky. This mouse has a Cre recombinase flanked by two mutated estrogen receptors and is driven by a human skeletal actin promoter. Past work confirmed the efficacy of this mouse for muscle-specific gene recombination in adult mice [21]. The tamoxifen inducible Cre recombinase mouse was crossed with the *Bmal1*-floxed mouse acquired through Jackson Labs (B6, 1294(Cg)-*Arntl*^tm1Weit/J^), to generate the inducible skeletal muscle-specific *Bmal1*-floxed mouse (iMS*Bmal1*^fl/fl^). Activation of Cre recombination was done by intraperitoneal injections of tamoxifen (2 mg/day) for five consecutive days when the mice reached 12 weeks. This age is chosen to eliminate any effects that the lack of *Bmal1* might have on skeletal muscle development and the rapid growth that occurs during post-natal maturation. Controls were vehicle (15 % ethanol in sunflower seed oil) treated with Cre^+/−^:*Bmal1*^*flox/flox*^ mice. Recombination specificity was confirmed and is demonstrated in Hodge et al. [13]. Prior to experimentation, mice were housed in 14:10, light-to-dark conditions. All experiments were performed 3–5 weeks post-treatment. Echo-MRI was also performed at 10–12 weeks post-treatment. Both male and female mice were used in this study. Mice were euthanized prior to glucose uptake experiments and tissue collection. Euthanasia was performed using anesthesia followed by cervical dislocation.

### Echo-MRI

Body composition was quantified in conscious mice at both 3–5 and 10–12 weeks post-treatment using EchoMRI Quantitative Magnetic Resonance Body Composition Analyzer (Echo Medical Systems, Houston, Texas) (mouse numbers *n* = 6 female iMSBmal1^+/+^ to *n* = 7 male iMSBmal1^+/+^ to *n* = 8 female iMSBmal1^−/−^: *n* = 10 male iMSBmal1^−/−^).

### Glucose tolerance tests and blood work

Glucose tolerance tests [*n* = 8/group (two females and six males) in both groups] were performed 2 h after lights-off (ZT14), a time at which glucose tolerance is highest [18]. Mice were fasted 6 h prior to the start of the test, and all measurements were done with an AlphaTRAK glucometer (Abbott Animal Health). A measurement was taken prior to injection of a bolus of glucose (0 min). Mice were injected by intraperitoneal injection with 2 mg/kg glucose, and additional measurements were taken at 15, 30, 60, 90, and 120 min post-injection. All fasting blood measures were obtained after a 6-h fast. Fasting blood glucose (*n* = 8/group (2 females and 6 males in both groups)) was measured with the AlphaTRAK glucometer, and fasting insulin (n = 7/group (3 females and 4 males in both groups)) was measured by collecting blood from the tail vein of the mice and running a colorimetric ELISA (Crystal Chem). Non-fasting blood glucose was measured by measuring blood glucose every 4 h for 24 h and averaged [*n* = 4 per time point; CT18 iMSBmal1^+/+^*n* = 2 F/2 M; iMSBmal1^−/−^*n* = 2 F/2 M; CT22 iMSBmal1^+/+^*n* = 4 M; iMSBmal1^−/−^*n* = 2 F/2 M; CT26 iMSBmal1^+/+^*n* = 2 F/2 M; iMSBmal1^−/−^*n* = 1 F/3 M; CT30 iMSBmal1^+/+^*n* = 1 F/3 M; iMSBmal1^−/−^*n* = 1 F/3 M; CT34 iMSBmal1^+/+^*n* = 1 F/3 M; iMSBmal1^−/−^*n* = 1 F/3 M]

### Glucose uptake experiments

All solutions in this experiment were bubbled with 95 % O_2_/5 % CO_2_ and kept at 35 °C. Extensor digitorum longus (EDL) muscles were excised from each leg of the mice [*n* = 8 (2 F/6 M)/group for insulin stimulation; *n* = 5 all females/group for 5-aminoimidazole-4-carboxamide ribonucleotide (AICAR) stimulation] and placed in a recovery media (Krebs Henseleit Buffer (KHB) with 0.1 % bovine serum albumin, 2 mM sodium pyruvate, 6 mM mannitol) with or without 2000 μU/mL insulin or 2 mmol/L AICAR. After 30 min, muscles were transferred to an incubation buffer (KHB with 0.1 % bovine serum albumin, 2 mM sodium pyruvate, 6 mM mannitol, 1 mM 2-deoxy-d-glucose, 2.25 μL/mL [3H]-2-deoxyglucose, 2 μL/mL [14C]-mannitol) with or without 2000 μU/mL insulin or 2 mmol/L AICAR (consistent with what the muscle was exposed to in recovery media) for exactly 20 (insulin) or 40 (AICAR) min. Following this incubation, tissues were flash frozen. Tissue lysates were prepared, and 100 μL of each sample was added to a scintillation vial with 5 mL of scintillation fluid followed by radioactivity measurement in a scintillation counter.

### Real-time PCR (RT-PCR)

RNA was isolated from gastrocnemius (GTN) samples of iMS*Bmal1*^+/+^ and iMS*Bmal1*^−/−^ mice [*n* = 7 (3 F/4 M)/group]. Briefly, 50–100 mg of GTN tissue was homogenized in 1 mL Trizol (Invitrogen). Phase separation, RNA precipitation and RNA washes, and RNA resuspension were carried out as per manufacturer’s instructions. RNA was quantified through spectrophotometrically (*λ* = 260 nm). Total RNA was then used to synthesize cDNA using a mixture of oligo(dT) primer and random hexamers in SuperScript III First-Strand Synthesis SuperMix (Invitrogen, Waltham, MA, USA) kit. Expression of glucose transporter type 4 (*Glut4*), hexokinase 2 (*Hk2*), and phosphofructokinase 1 (*Pfk1*) was performed using cDNA and the following Taqman primers (Applied Biosystems): Mm01245502_m1, Mm00443385_m1, Mm01309576_m1, Mm99999915_g1, and Mm02343715_g1. RPL26 or glyceraldehyde 3-phosphate dehydrogenase (GAPDH) were used as the internal calibration controls. The ∆∆CT method was used for the quantification of real-time PCR data. Gene expression in each sample was shown as the relative value compared to the mean vehicle value in that tissue (GTN).

### Western blot analysis

Tissue lysates were made from GTN muscles of iMS*Bmal1*^+/+^ and iMS*Bmal1*^−/−^ mice [*n* = 7 (3 F/4 M)/group]. Proteins were separated by SDS-PAGE using 4–15 % Tris-HCl precast gels (BioRad), transferred, and immunoblotted using routine methods. GLUT4 was detected using a primary monoclonal GLUT4 antibody (Cell Signaling, #2213) and an AlexaFluor680 goat anti-rabbit secondary antibody (Invitrogen, #A-21109).

### Hexokinase activity assay

Hexokinase activity was measure following as previously described [30]. GTN tissue [*n* = 7 (3 F/4 M)/group] was homogenized in a buffer (1:10, weight to volume) containing 150 mM KCl, 10 mM MgCl_2_, 5 mM EDTA, and 5 mM β-mercaptoethanol. Samples were centrifuged at 15,000×*g* for 1 h while experimental solutions A (47 mM Tris (pH 7.4), 10 mM MgCl_2_, 0.8 mM NADP, 0.5 mM glucose, 5.0 mM mercaptoethanol, 0.1 units glucose 6-phosphate dehydrogenase) and B were prepared(47 mM Tris (pH 7.4), 10 mM MgCl_2_, 0.8 mM NADP, 0.5 mM glucose, 5.0 mM mercaptoethanol, 0.1 units glucose 6-phosphate dehydrogenase, 5 mM ATP, 0.27 mM phosphoglyceric acid). An Eppendorf tube containing 2.45 mL of A or B was made for each tissue sample. After centrifugation was complete, 0.05 mL of tissue sample was added to each tube. Absorbance was measured at 30 °C and 340 nM every 2 s for 10 min. Samples were measured in duplicates. As the glycolytic reaction takes place, NADP is oxidized to form NADPH which has an absorbance at 340 nm.

### Phosphofructokinase activity assay

The same tissue samples [*n* = 7 (3 F/4 M)/group] used for the hexokinase activity assay were used for the phosphofructokinase assay. The reaction mixture for this assay contained 50 mM Tris-HCl (pH 8), 1 mM EDTA, 6 mM MgCl_2_, 2.5 mM dithiothreitol, 0.16 mM NADH, 1 mM ATP, 1 mM fructose-6-phosphate, 0.4 units aldolase, 2.4 units triose-phosphate isomerase, and 0.4 units α–glycero-phosphate dehydrogenase. Five microliters of tissue sample was added to 295 μL of reaction mixture, and absorbance was read at 25 °C and 340 nM for 10 min. As the glycolytic reaction takes place, NADH, which is detectable at 340 nm, is reduced to NAD^+^ and absorbance decreases.

### Metabolomics data

GTN muscles were dissected and freeze clamped in situ at ZT14 from anesthetized iMS*Bmal1*^+/+^ and iMS*Bmal1*^−/−^ mice [*n* = 7 (3 F/4 M)/group] at 5 weeks post-treatment. Flash-frozen samples were sent to the University of Michigan Metabolomics Core Services for targeted metabolomics of glycolysis/tricarboxylic acid (TCA)/nucleotides involved in central metabolism. Twenty milligrams of skeletal muscle tissue was extracted first exposing the tissue to liquid nitrogen to harden the sample, then grinding the tissue in a pre-chilled mortar and pestle until the tissue was deemed adequately disrupted. The ground tissue was then transferred to a microtube, and metabolites were extracted with 0.5 mL of a mixture of methanol, chloroform, and water (8:1:1) containing isotope-labeled internal standards using a probe sonicator at 40 % output power, 20 % duty cycle for 20 s. Samples were allowed to rest at 4 °C for 10 min and then centrifuged at 4 °C, 14,000 rpm for 10 min. The extracts were removed and placed into an autosampler vial for mass spec analysis. Ten microliters of each sample was removed and pooled in a separate autosampler vial for quality control purposes. A series of calibration standards were prepared along with samples to quantify metabolites.

LC-MS analysis was performed on an Agilent system consisting of a 1260 UPLC module coupled with an 6520 Quadrupole-Time-of-flight (QTOF) mass spectrometer (Agilent Technologies, CA.) Metabolites were separated on a 150 × 1 mm Luna NH2 hydrophilic interaction chromatography column (Phenomenex, CA) using 10 mM ammonium acetate in water, adjusted to pH 9.9 with ammonium hydroxide, as mobile phase A, and acetonitrile as mobile phase B. The flow rate was 0.075 mL/min, and the gradient was linear 20 to 100 % A over 15 min, followed by isocratic elution at 100 % A for 5 min. The system was returned to starting conditions (20 % A) in 0.1 min and held there for 10 min to allow for column re-equilibration before injecting another sample. The mass spectrometer was operated in ESI-mode with the following conditions: gas temperature 350 °C, drying gas 10 L/min, nebulizer 20 psi.

Data were processed using MassHunter Quantitative analysis version B.07.00. Metabolites in the glycolysis/tricarboxylic acid (TCA)/ppp pathways were normalized to the nearest isotope-labeled internal standard and quantitated using two replicated injections of five standards to create a linear calibration curve with accuracy better than 80 % for each standard. Other compounds in the analysis were normalized to the nearest internal standard, and the peak areas were used for differential analysis between groups. Results from this analysis were used to identify enriched pathways using the online software Metaboanalyst 3.0, a comprehensive tool for metabolomic data analysis supported by The Metabolomics Innovation Center (TMIC), a Genome Canada-funded core facility.

### Activity recordings

Telemetry (Data Sciences International (DSI)) was used to evaluate changes in cage activity after vehicle/tamoxifen injection. Mice were anesthetized with isoflurane, and transmitter units (PhysioTel PA-C10; DSI) were implanted. Mice were housed singly and allowed to recover for 1 week. Data were recorded for 24 h/day, for 3–4 days weekly up to 10 weeks after vehicle or tamoxifen treatment. Data were collected and analyzed with DSI Dataquest ART4.1 telemetry software.

### Statistics

For statistics on blood concentrations, glucose tolerance, RT-PCR, body composition, behavior and enzyme activities, a Student’s *t* test was utilized. Two-way ANOVA was used to sex differences in body weight and body composition and data from glucose uptake experiments (comparing time exposed to insulin and genotype). Statistical analysis of metabolites was largely performed using Student’s *t* test to address differences between genotypes. To address whether metabolites within the glycolytic pathway were changed we used Hotelling’s T-squared test to determine if metabolites were changed between iMS*Bmal1*^+/+^ and iMS*Bmal1*^−/−^ mice.

## Results

**Fig. 1:**
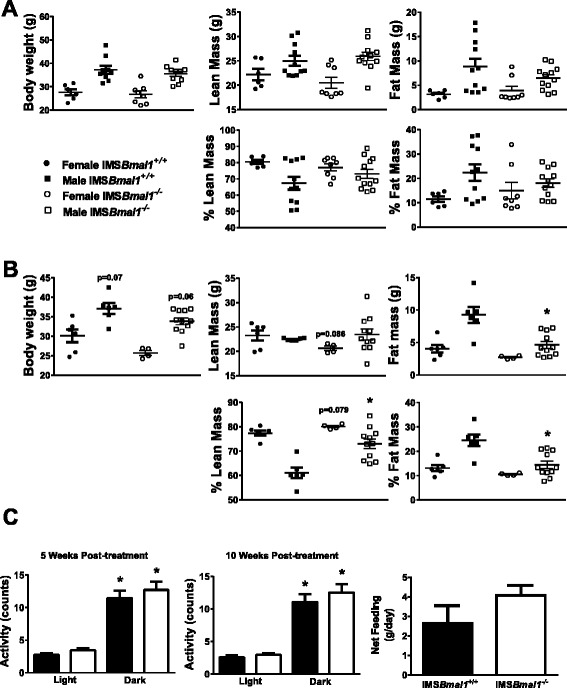
Body composition but not behavior was altered in the iMSBmal1^−/−^ mice. **a** Body weight and composition (lean and fat mass) at 3–5 weeks post-treatment (*n* = 17 iMSBmal1^+/+^, *n* = 20 iMSBmal1^−/−^). **b** Body weight and composition (lean and fat mass) at 10–12 weeks post-treatment (*n* = 12 iMSBmal1^+/+^, *n* = 15 iMSBmal1^−/−^). **c** Feeding (4 days) at 5 weeks post-treatment (*n* = 4 iMSBmal1^+/+^, *n* = 6 iMSBmal1^−/−^) and activity (3 days) at 5 and 10 weeks post-treatment (*n* = 5 iMSBmal1^+/+^, *n* = 5 iMSBmal1^−/−^)

Previous studies examining the germline *Bmal1* knock out mouse demonstrated significant changes in body weight and systemic metabolic parameters such as the glucose tolerance test [26]. However, in those studies, *Bmal1* was knocked out in all tissues during embryonic development. Here, we focus on the loss of *Bmal1* in the skeletal muscle of the adult mouse. For this study, we generated an inducible skeletal muscle-specific *Bmal1* knock out mouse (iMS*Bmal1*^*−/−*^; following tamoxifen treatment) for the targeted disruption of the molecular clock mechanism only in adult skeletal muscle [13]. We performed the tamoxifen/vehicle injection at 12–14 weeks of age to avoid the potential problem with fusion of non-recombined satellite cells during the maturational, rapid growth phase of skeletal muscle from 6 to 10 weeks of age. Using this approach, we found that at 3–5 weeks post-treatment, there was no statistical difference in body weight or body composition between the two groups (male or female) (Fig. 1a). However, by 10–12 weeks post-treatment, the iMS*Bmal1*^*−/−*^ mice had significantly lower body weights (Fig. 1b). Two-way analysis of variance determined that there was no significant interaction between sex and treatment, but there were significant sex (*p* < 0.0001) and treatment (*p* = 0.0079) effects with males exhibiting the greatest loss in body weight. Fat mass was also significantly lower in the iMSBmal1^−/−^ mice and, while there were differences between males and females (*p* = 0.002), there was also a significant treatment effect (0.0012) with no significant interaction. When analyzing the lean mass data, there were no statistically significant changes in lean mass either due to sex or to treatment. Not surprisingly, the iMSBmal1^−/−^ mice were leaner with a lower percent fat (18.83 ± 2.2, iMS*Bmal1*^+/+^; 13.41 ± 1.2, iMS*Bmal1*^−/−^) (Fig. 1b). These results indicate that the primary change in body weight for both male and female mice is through changes in body fat but that males exhibited a significantly greater change. It is important to note that these changes in body composition could not be attributed to altered feeding or behavior, as there were no detectable differences in net feeding obtained from metabolic cage analysis in the iMS*Bmal1*^−/−^ mice or net activity obtained from telemetry monitoring at 5 and 10 weeks post-treatment (Fig. 1c).

**Fig. 2:**
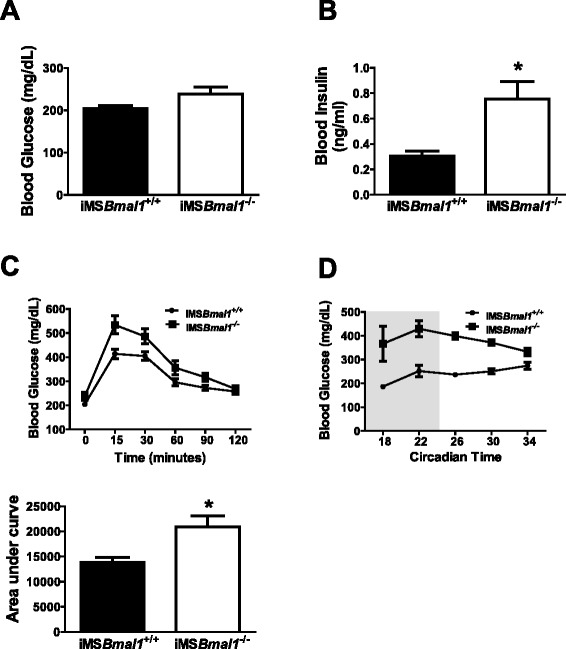
iMSBmal1^−/−^ mice display altered systemic glucose handling*.* **a** Fasted blood glucose (*n* = 8/group (2 females and 6 males in both groups)). **b** Fasted blood insulin *n* = 7/group (3 females and 4 males in both groups). **c** Glucose tolerance depicted as blood glucose versus time post-glucose injection and area under the curve (*n* = 8/group (2 females and 6 males) in both groups). **d** Non-fasted blood glucose measured every 4 h for 24 h [*n* = 4 per time point: CT18 iMSBmal1^+/+^ *n* = 2 F/2 M; iMSBmal1^−/−^ *n* = 2 F/2 M; CT22 iMSBmal1^+/+^ *n* = 4 M; iMSBmal1^−/−^ *n* = 2 F/2 M; CT26 iMSBmal1^+/+^ *n* = 2 F/2 M; iMSBmal1^−/−^ *n* = 1 F/3 M; CT30 iMSBmal1^+/+^ *n* = 1 F/3 M; iMSBmal1^−/−^ *n* = 1 F/3 M; CT34 iMSBmal1^+/+^ *n* = 1 F/3 M; iMSBmal1^−/−^ *n* = 1 F/3 M]

We next performed glucose tolerance tests, as well as, assessed blood glucose and insulin levels. Fasting blood glucose (Fig. 2a) was not significantly different between groups [*n* = 7 (3 F/4 M)/group], but iMS*Bmal1*^−/−^ mice had significantly elevated fasting insulin levels (2-fold higher) compared to iMS*Bmal1*^+/+^ mice (Fig. 2b). When injected with a bolus of exogenous glucose, iMS*Bmal1*^−/−^ mice exhibited a greater overall increase in blood glucose. In addition, glucose levels remained elevated for a longer period of time in the iMS*Bmal1*^*−/−*^ mice. The combination of the overall increase in blood glucose, and the extended time that blood glucose remained elevated in the iMS*Bmal1*^−/−^ mice, significantly increased the area under the curve (51.9 % increase) when compared to the control mice (iMS*Bmal1*^+/+^) (Fig. 2c). None of the tests on this cohort of mice showed any difference in response within a genotype for the male (*n* = 4) vs. female (*n* = 3) mice. Analysis of blood glucose levels was also taken from non-fasted iMS*Bmal1*^*+/+*^ and iMS*Bmal1*^*−/−*^ mice during a circadian time course collection, every 4 h over a 28-h period [13]. In this set of experiments, we found that iMS*Bmal1*^−/−^ mice displayed significantly higher blood glucose values (~42 %) across every time point collected relative to iMS*Bmal1*^+/+^ mice. We used both male and female mice for these experiments (see “Methods” section for detailed description), and we did not detect any difference in non-fasted blood glucose between either sexes. These results are plotted for each group, at each time point in Fig. 2d.

**Fig. 3:**
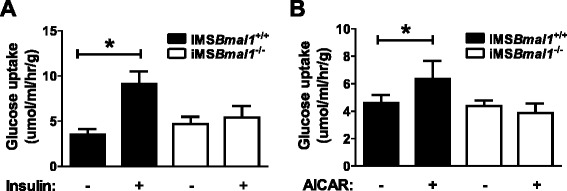
Glucose uptake was diminished in the iMSBmal1^−/−^ mice*.* **a** Insulin-stimulated glucose uptake in the EDL muscles using a maximum insulin dose and 20 min of exposure [*n* = 8 (2 F/6 M)/group]. **b** AICAR-stimulated glucose uptake in the EDL muscles after 40 min of exposure incubation media with or without AICAR *n* = 5 (5 F)/group

Skeletal muscle is the major site for glucose disposal in the postprandial state. The measured glucose intolerance, elevated fasting insulin levels, and elevated non-fasting blood glucose levels implied a defect in skeletal muscle glucose uptake. We examined this further by testing whether the loss of *Bmal1* in the skeletal muscle alters either insulin-stimulated or AICAR-stimulated glucose uptake pathways in the EDL muscles of iMS*Bmal1*^*+/+*^ and iMS*Bmal1*^*−/−*^ mice. We found that there was no difference in basal levels of glucose uptake but that the insulin-stimulated glucose uptake was significantly impaired in the iMS*Bmal1*^−/−^ mice (Fig. 3a). In addition, we found that when EDL muscles were treated with AICAR (*n* = 5 females/group), glucose uptake increased significantly in the iMS*Bmal1*^*+/+*^ muscle but, like seen with insulin, there was no change in glucose uptake in the EDL of the iMS*Bmal1*^−/−^ mice (Fig. 3b).

**Fig. 4:**
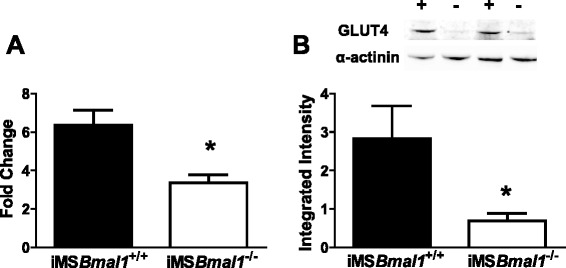
*Both mRNA and protein expression of the glucose transporter type 4 (Glut4/GLUT4) were decreased in the iMSBmal1* ^*-/-*^ *mice.* **a** mRNA expression of *Glut4* in the GTN muscle measured using RT PCR. (n = 6). **b** Protein content of GLUT4 in the GTN muscle was measured as integrated intensity of the fluorescent bands (n = 7)

Insulin and AICAR stimulate separate pathways to facilitate glucose uptake [8]. However, our results are consistent with a model in which the loss of *Bmal1* in the skeletal muscle leads to altered expression of a common component to both pathways. We focused on the levels of *Glut4*, and we found that in the iMS*Bmal1*^−/−^, mRNA expression is reduced by 47.2 % and protein content of the glucose transporter was reduced by 75.5 % relative to that measured in iMS*Bmal1*^+/+^ mice (Fig. 4a, b).

**Fig. 5:**
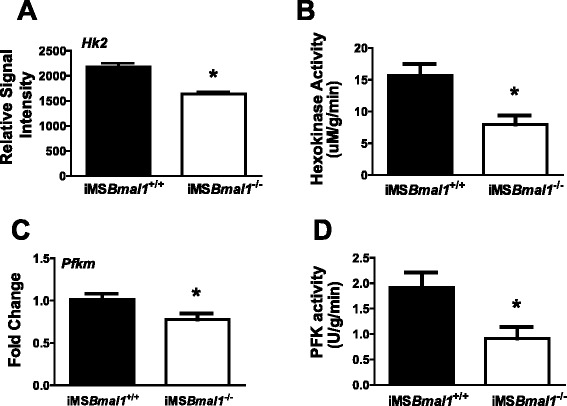
Activity of rate-limiting glycolytic enzymes (HK2 and PFK1) was significantly decreased in the iMSBmal1^−/−^ mice. **a** mRNA expression of *Hk2* obtained from microarray data (*n* = 6). **b** Enzymatic activity of the rate-limiting enzyme HK2 in the GTN muscle measured using a spectrophotometric assay (*n* = 7). **c** mRNA expression of the rate-limiting enzyme *Pfkm* in the GTN muscle (*n* = 7). **d** Enzymatic activity of PFKM in GTN muscles

Upon entering the muscle cell, glucose is phosphorylated to glucose-6-phosphate by the glycolytic enzyme HK2. This rapid transition of glucose to glucose-6-phosphate allows the cell to maintain a glucose concentration gradient relative to the extracellular fluid to help sustain glucose influx when GLUT4 is present at the membrane. In this regard, it has been proposed that HK2 activity can modulate glucose uptake. We analyzed GTN muscle microarray data from our circadian dataset [13]. Signal intensity measured for *Hk2* mRNA was significantly lower in the iMS*Bmal1*^*−/−*^ mice (Fig. 5a). We next tested whether there was any change in the enzyme activity of HK2 in the GTN of the iMS*Bmal1*^*+/+*^ and iMS*Bmal1*^*−/−*^ mice (*n* = 3 females/4 males for each group) and consistent with the decreased expression, we found that HK2 activity (uM/g/min) was 49.5 % reduced in muscle extracts from iMS*Bmal1*^−/−^ mice (Fig. 5b). Besides having an impact on glucose uptake, HK2 is a rate-limiting enzyme in the glycolytic pathway. In order to further evaluate glycolytic flux in the iMS*Bmal1*^*−/−*^ mice, we examined another rate-limiting enzyme of glycolysis, phosphofructokinase (*Pfk1*). Real-time PCR results confirmed that *Pfk1* mRNA expression was reduced by 23.3 % in iMS*Bmal1*^−/−^ mice, and like HK2, we found that enzyme activity for *Pfk1* was significantly reduced by 52.4 % (Fig. 5c, d).

To gain a better sense of metabolite changes in the iMS*Bmal1*^*−/−*^ skeletal muscles, we used a targeted metabolomics approach to evaluate glycolytic/TCA-related metabolites in the GTN muscles the mice [*n* = 7 ((3 females/4 males)/group)]. All skeletal muscles were flash frozen in situ with tongs cooled to liquid N_2_ and shipped to the Michigan Regional Comprehensive Metabolomics Resource Core for glycolytic and TCA cycle metabolites. The results of these assays are provided in Table 1. We found that glucose levels were significantly elevated within the muscle of the iMS*Bmal1*^−/−^ mice compared to the iMS*Bmal1*^+/+^ mice. In addition, we measured glycogen using a biochemical assay and found that glycogen was also significantly increased in the GTN muscles of the iMS*Bmal1*^*−/−*^ mice (Table 1). When considered in context with the decreased *Pfk1* activity, this suggests that glucose is being preferentially stored vs. oxidized in the skeletal muscle. We also found that metabolites of glycolysis, including glyceraldehyde 3-phosphate and 2- and 3-phopshoglycerate, were significantly lower and phosphoenolpyruvate trended to be lower in the iMS*Bmal1*^−/−^ mice. Interestingly, levels of lactate were also significantly elevated in the GTN muscle of iMS*Bmal1*^−/−^ mice.

**Table 1:**
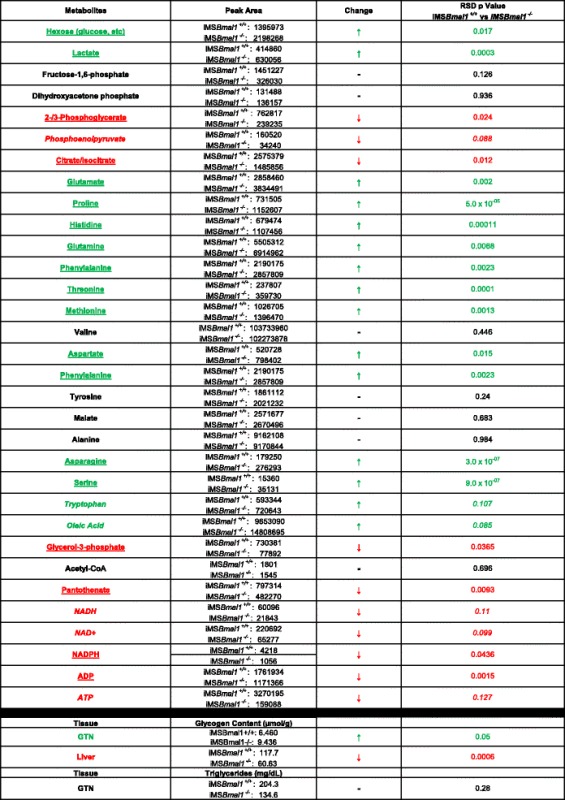
List of metabolites involved in glycolysis, fat oxidation, and TCA cycle in iMS*Bmal1* ^*+/+*^ and iMS*Bmal1* ^*−/−*^ mice 5 weeks post-treatment [*n* = 7 (3 F/4 M)/group]

Texts in green are upregulated, *p* < 0.05; red downregulated, *p* < 0.05; green italicized trending up; red italicized trending down; and black no change

**Fig. 6:**
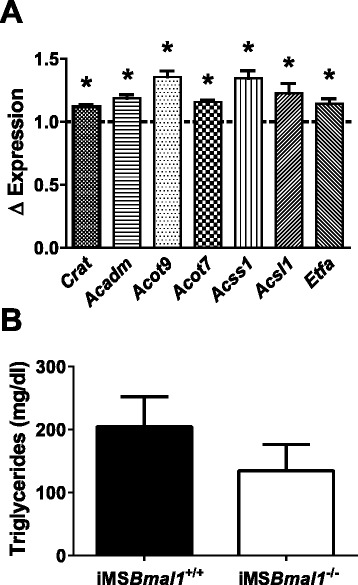
Genes involved in fat metabolism are upregulated in the iMSBmal1^−/−^ GTN*.* **a** Array data revealed that genes important in fat metabolism in the skeletal muscle are significantly upregulated in the iMS*Bmal1* ^*−/−*^ GTN. **b** Triglyceride levels trended toward a decrease in the iMS*Bmal1* ^*−/−*^ GTN

**Fig. 7:**
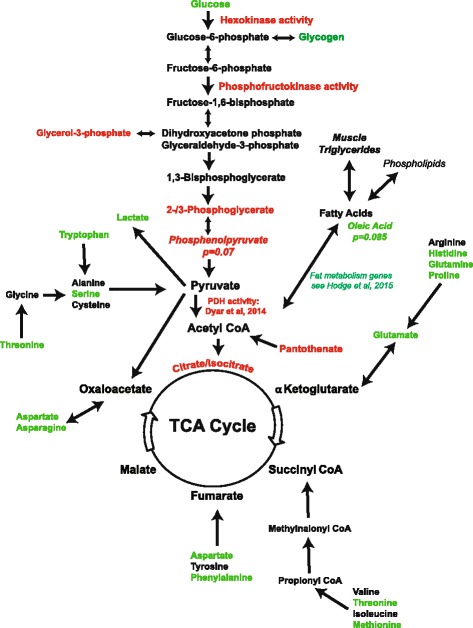
Summary of changes linked to substrate oxidation in the skeletal muscle. Metabolomics data, enzymatic activity data, glycogen data, and triglyceride data are included. *Green* upregulated, *red* downregulated, *green italicized* trending up, *red italicized* trending down, and *black* no change

In addition to changes in glucose and glycolytic intermediates, the metabolomics data revealed significant changes in a number of amino acids that are known to have functions associated with the maintenance of TCA cycle intermediates. The observed changes in metabolites in glycolysis and amino acids suggested we also examined genes associated with fat metabolism. Analysis of our microarray data [13] demonstrated an increase in several genes associated with fat metabolism (Fig. 6a). We did not detect any significant changes in triglyceride levels in the GTN, but levels of oleic acid trended toward lower (*p* = 0.085) and levels of pantothenate (vitamin B_5_), which is used to synthesize coenzyme A (CoA), were significantly down (*p* = 0.0093). The list of the amino acid changes is provided in Table 1, and we have integrated all the metabolomics data with potential impact on glycolysis and the TCA cycle as a summary figure (Fig. 7).

## Discussion

Skeletal muscle is a highly metabolic tissue and is primarily responsible for the disposal of glucose in the postprandial state [6]. Circadian rhythms play an important role in regulating metabolism in a tissue-specific manner, and recent research demonstrates that disruption of circadian rhythms leads to metabolic dysfunction and disease [5, 13, 31, 34]. Our understanding of the function of the clock and core molecular clock genes, such as *Bmal1*, in skeletal muscle is still quite elementary. In our investigation, we demonstrate that loss of the core circadian clock gene, *Bmal1*, solely in adult skeletal muscle quickly manifests in disrupted glucose metabolism as demonstrated by impaired glucose uptake with a concomitant reduction in the muscle glucose transporter GLUT4 and decreased activities of key rate-limiting enzymes of glycolysis. Disruption of the molecular clock also led to changes in metabolites within the TCA cycle (citrate to isocitrate), and significant increases in amino acids and genes that regulate fat metabolism pointing to altered substrate metabolism for energy and maintenance of TCA cycle intermediates. Lastly, our findings demonstrate the contribution of disrupted skeletal muscle metabolism to system homeostasis including hyperglycemia in the non-fasting condition, glucose intolerance, and altered body composition in the absence of feeding or physical activity changes.

Our work demonstrates that loss of *Bmal1* only in the skeletal muscle can significantly affect glucose uptake into the skeletal muscle and implicates that these effects of *Bmal1* are likely mediated through regulation at least two different levels. First, we found that both insulin-stimulated glucose uptake and AICAR-stimulated glucose uptake are diminished with loss of *Bmal1* in the skeletal muscle. While these two signaling pathways for glucose uptake are distinct, the end target, GLUT4, is common and is significantly downregulated at the protein level [1, 5, 28]. In addition, we found that the enzyme activity for HK2 was almost 50 % lower compared to control levels. This is consistent with our findings of higher free glucose levels in the cell and would, in turn, function to decrease the concentration gradient between the inside and the outside of the muscle cell and thus, function to lower the rate of facilitated transport via GLUT4. In a study by Fueger et al. in 2003, they demonstrated that partial *Hk2* knockdown was enough to result in a decrease in exercise-stimulated glucose uptake [9]. We also found that one of the primary glycolytic rate-limiting enzymes, *Pfk1* [3, 22], was reduced at the mRNA level with an even more dramatic decrease in enzyme activity in the iMS*Bmal1*^−/−^ mice. Lowered *Pfk1* activity coupled with observations of lowered pyruvate dehydrogenase (PDH) activity by Dyar et al. [5] provides strong evidence for a significantly lower flux through glycolysis. This change in glycolytic flux has been proposed to have a secondary impact on glucose uptake by muscle [25]. Recent studies have also demonstrated the potential for a confounding effect due to tamoxifen treatment on metabolic parameters. In the most recent study, Hesselbarth et al. demonstrated transient changes in fat mass with a significant decrease at 1 week followed by a significant increase at 5 weeks post-treatment in C57BL/6NTac male mice [12]. These findings are very different compared to our results in which the tamoxifen-treated mice, male and female, exhibit no early change in body fat but are significantly leaner 10 weeks. The fact that our mice are leaner, and not fatter, at the 10 weeks time period strongly supports our conclusion that loss of *Bmal1* in muscle is the upstream mediator of the changes in systemic body composition.

Using a targeted metabolomic approach, we were also able to show that loss of *Bmal1* only in adult skeletal muscle is sufficient to induce significant decreases in metabolites within the glycolytic pathway and TCA cycle with concomitant increases in selective amino acids that can function to support the TCA cycle intermediates. Decreased levels of 2-/3-phosphoglycerate and glycerol 3-phosphate in combination with trending decreases in phosphoenolpyruvate are consistent with a decreased glycolytic flux and lower PFK1 activity. The work from Dyar et al. [5] showed that rates of PDH activity were lower in muscle of *Bmal1* knockout mice so it was not surprising to find that levels of lactate were elevated even in the context of decreased glycolytic flux. With the exception of a reduction in citrate/isocitrate, we did not see changes in most TCA intermediates (e.g., oxaloacetate, α-ketogluterate, and fumerate) but many amino acids were elevated suggesting that proteins were being metabolized to support the need for pyruvate, oxaloacetate, α-ketogluterate, fumerate, and succinate. Amino acids are linked to the TCA cycle through certain TCA cycle intermediates. If TCA cycle intermediates are present in sufficient amounts, they can serve as precursors for the synthesis of amino acids. In contrast, when energy production from glucose oxidation is compromised and TCA cycle intermediate levels become vulnerable, this can lead to increased protein degradation to provide amino acids into the TCA cycle. As seen in Fig. 6, the amino acids that changed are all significantly higher in iMS*Bmal1*^−/−^ mice. Since multiple lines of evidence suggest that glucose oxidation is blunted in iMS*Bmal1*^−/−^mice, it is probable that the skeletal muscle is relying on alternate energy sources such as protein and fat. The fact that citrate and isocitrate in iMS*Bmal1*^−/−^ GTN are reduced supports a shift in substrate utilization. In healthy muscle, pyruvate generated from glycolysis may enter the TCA cycle as acetyl CoA at oxaloacetate and then the TCA cycle proceeds [23]. Citrate and isocitrate are products generated during the initial steps of the TCA cycle. Glucose oxidation would generate these while fatty acid and amino acid oxidation enter the TCA cycle downstream of these events.

In light of the fact that skeletal muscle, a vital metabolic tissue, cannot efficiently dispose of glucose and utilize it as a fuel source, one can expect that this would impact overall metabolic health. While remarkable that loss of a single gene is the skeletal muscle is sufficient to alter systems metabolism, it is not unprecedented as muscle-specific *Glut4* knockout mice gain weight more slowly and have reduced fat stores [37]. This change in glucose metabolism in skeletal muscle likely drives oxidation of fat in adipose tissue resulting in leaner mice. This same study and some studies utilizing hexokinase- and phosphofructokinase-deficient mice demonstrate development of insulin resistance, glucose intolerance, and elevated blood insulin with disruption of glycolytic flux [9, 22, 37].

## Conclusions

The findings of this study highlight the novel role(s) for skeletal muscle *Bmal1* in regulating tissue glucose metabolism (i.e., decreased GLUT4 and glycolytic enzymes), substrate utilization (i.e., changes in citrate to isocitrate within the TCA cycle), as well as system homeostasis (i.e., hyperglycemia in the non-fasting condition, glucose intolerance, and altered body composition). However, there is still much that is unknown. Further research is required to elucidate the exact mechanism by which *Bmal1* alters GLUT4 content, glucose uptake, and glycolytic gene expression and activity in the skeletal muscle. We demonstrate that skeletal muscle *Bmal1* is critical for skeletal muscle carbohydrate metabolism and disruptions in glucose oxidation, and subsequent compensations impact overall metabolic health. Targeting the molecular clock mechanism provides a novel approach for future treatment modalities in the treatments of diabetes and metabolic disease.

## Abbreviations

2PG: 2-phosphoglycerate
3PG: 3phosphoglycerate
AICAR: 5-aminoimidazole-4-carboxamide ribonucleotide
*Bmal1*: brain and muscle Arnt-like 1
*Clock*: circadian locomotor output cycles kaput
*Cry1/2*: cryptochrome 1 and 2
DHAP: dihydroxyacetone phosphate
EDL: extensor digitorum longus
FBP: fructose 1,6-bisphosphate
GAPDH: glyceraldehyde 3-phosphate dehydrogenase
*Glut4*: glucose transporter type 4
*Hk2*: hexokinase 2
iMS*Bmal1*^−/−^: inducible skeletal muscle-specific *Bmal1* knockout mice (tamoxifen treated)
iMS*Bmal1*^+/+^: inducible skeletal muscle-specific *Bmal1* positive mice (vehicle treated)
iMS*Bmal1*^fl/fl^: inducible skeletal muscle-specific *Bmal1* floxed mice
PDH: pyruvate dehydrogenase
*Per*1/2: period 1 and 2
*Pfk1*: phosphofructokinase 1
*Rpl26*: ribosomal protein 26
